# Outcome of Cochlear Implantation in Post-Meningitis Deaf Children

**DOI:** 10.5812/ircmj.3394

**Published:** 2013-01-05

**Authors:** Mahdiyeh Hasanalifard, Mohammad Ajalloueyan, Susan Amirsalari, Amin Saburi

**Affiliations:** 1 New Hearing Technologies Research Center, Baqiyatallah University of Medical Sciences, Tehran, IR Iran

**Keywords:** ENT, Pediatrics, Pediatrics Surgery

Dear Editor,

Cochlear implantation (CI) is an effective procedure for treatment of children with severe to profound Sensori-neural hearing loss (SNHL). In spite of suitable outcome in many patients, choosing the candidates should be regarding to the child age and etiology (congenital or acquired SNHL) (1-4). Bacterial meningitis (BM) is one of the most common etiologies of acquired SNHL which estimated approximately 60 - 90% of all cases of secondary SNHL at children (5). Due to concomitant neurological sequelae such as seizure, visual impairment and hydrocephalus, the successful outcome of CI in these cases remained doubtful. We introduce a case series about outcome of cochlear implantation at children with SNHL due to BM as the preliminary report from Iran. Two hundred eighty-four children with hearing loss presenting to the cochlear implantation center of the Baqiyatallah Hospital between 2008 and 2010 were evaluated and finally, eight children with Post Meningitis deafness (PMD) were enrolled. Profound SNHL was confirmed based on the average of pre-implantation unaided pure-tone thresholds over 90 dB. There were complementary investigations for overruling other cause of SNHL. The Nucleus 22 channel device and a speech processors device was used, routinely although other option has been considered in special subjects. Each cases assessed by Nerve Response Telemetry (NRT) intra-operatively and 45 days after surgery. Speech Intelligibility Rating (SIR) and Categories of Auditory Perception scale (CAP) tests was conducted in the best-aided situation both before and after implantation.6 All cases were assessed at three, six, 12, and 24 months after CI. This investigation was approved by the ethical review board. The mean age of children at the meningitis diagnosis was 15.75 ± 6.77 (Mean ± SD) months and the mean age at cochlear implantation was 31.12 ± 1.27 months. Two patients was male (patients number 1&4). The microorganism cultured from the CSF was identified in 3 (37.5%) patients. In three patients (No. 1, 3&4) the causative microorganism was Streptococcus Pneumoniae (*Pneumococcus*) and in other subjects causative microorganisms were unknown. Electrode insertion in 6 out of eight patients was complete but two children required cochlear drill-out and in one child short electrodes was used. There was no serious complication after operation during 6 months follow up. The mean of NRT at the baseline, 3 and 6 months later was 69.37 ± 96.78, 187.37 ± 19.24 and 184.62 ± 17.32, respectively (Table 1). We used SPSS version 16 and repeated measured ANOVA test to compare the CAP and SIR findings. By using this test we were able to compare the CAP and SIR score between more than two stages (0, 3 and 6 month after implantation). Three months after CI, the mean score of CAP test developed from 0.62 ± 0.74 at the baseline to 3.00 ± 1.41 and also increased to 3.75 ± 1.16 at the 6-months after CI (P < 0.001). Also, SIR scored a mean of 1.25 ± 0.46 at the baseline improved to 1.37 ± 0.74 at 3 months after implantation (P = 0.351) and a mean of 2.25 ± 0.88 at 6 months later (P < 0.001) (Table 2). Previously, the CI success and efficacy in children with additional disability such as PMD compared to children with pure SNHL were debatable (6, 7). This supposition was because of having concomitant neurological squeal. The electrode may be inserted incompletely due to ossified cochlea (8), although, results of several previous studied were equivocal. Howard et al recommended that neurologic squeal of BM annoy the improvement of speech perception after CI in patients with PMD (9) El-Kashlan et al. showed children with cochlear ossification due to BM have significant lower speech perception improvement than a matched control children with congenital SNHL at both the 6 and 24-month follow-up after CI but with extended follow-up, some children with ossification had speech perception partially (10). Eshragi et al. revealed children with PMD and those with cochlear ossification who undergo CI may require frequent programming adjustments to obtain the optimal performance because levels of stimulation increase over the time (11). Partial insertion is more suitable and comfortable than complete insertion in ossified cochlea or labyrinth for surgeons (12). Age and causative microorganism are important factors to determinate the outcome in children with post meningitis deafness (13). Also, the role of time between PMD and implantation is arguable. Some survey recommended that CI should be performed after diagnosis of PMD as soon as possible and other suggested late approach (14).Young et al. showed that early bilateral simultaneous CI in children with PMD increases the likelihood of binaural hearing and ensures implantation of the better ear in this population of children whose course is often complicated by formation of scar tissue and ossification within the cochlea (15). Regarding to the results of present study and similar studied we conclude that children with post meningitis deafness could be Benefited from CI. However, Studies with larger sample size and a control group with longer follow-up period for confirming the prognostic factors are recommended.

**Table 1:** Nerve Response Telemetry (NRT) Findings

No.	Electrodes insertion	NRT (at the baseline)	NRT a (45 days after CI) a	NRT (3 months after CI)	NRT (6 months after CI)
** 1**	Suitable	0	205	195	194
** 2**	Suitable	155	145	140	143
** 3**	Drill & Short electrodes	0	201	196	197
** 4**	Suitable	0	196	194	190
** 5**	Suitable	195	186	190	190
** 6**	Drill & normal electrodes	0	195	196	193
** 7**	Suitable	205	187	195	185
** 8**	Suitable	0	195	193	185

^a^Abbreviations: CI, cochlear implantation; NRT, nerve response telemetry

**Table 2:** CAP and SIR Score in Patient Before and After CI

No.	CAP a(efore CI)	CAP (3 months after CI a)	CAP (6 months after CI)	SIR a (before CI)	SIR (3 months after CI)	SIR (6 months after CI)
** 1**	0	1	2	1	1	2
** 2**	2	6	6	2	3	4
** 3**	0	3	3	1	1	2
** 4**	0	3	4	1	1	2
** 5**	0	2	3	1	1	1
** 6**	** 1**	** 3**	4	1	1	2
** 7**	1	3	4	1	1	2
** 8**	1	3	4	2	2	3

^a^Abbreviations: CAP, Categories of Auditory Perception; CI, cochlear implantation; SIR, speech intelligibility rating
